# A Great Saving in Drugs

**Published:** 1907-05-04

**Authors:** 


### CORRESPONDENCE.

### A Great Saving in Drugs.

Hirudo writes: The institution of a R.M.O.'s department in your paper is a step which will certainly be appreciated. It has often been a dream of mine to see established a periodical devoted to the interests of this large class of practitioners—a paper which should be of real value, and should keep the provincial house surgeon in touch with his brethren. How often does it not happen that he would like to compare notes with a colleague about some little friction that has taken place, or to put forward some ingenious wrinkle which he has found of help to him in his work? Yes, there is room for something between the *Lancet* and the various students' gazettes, which, however amusing their personalities may be, are generally of no help to those who have left their Alma Mater.

The article in the issue for April 20, dealing with hospital residents and economy in the use of drugs, interests me not a little; in fact, I feel aggrieved, inasmuch as I had started to prepare a short note on the selfsame subject, only to find myself forestalled. Having had the opportunity before qualification of obtaining much practical experience of drugs, I was, perhaps, as a house surgeon, rather more interested in the dispensing department than is the average man. Your remarks on the subject of economy in drugs are most opportune, and I can fully bear out your points.

At a certain small provincial hospital with which I was connected I availed myself of the opportunity to test the proposition that, without sacrificing the patients in the slightest degree, a great saving in drugs can be effected. The average amount of the drug bill had been about £240 per annum, and there is no reason whatever to suppose that my predecessors had been particularly careless or extravagant. During the year in which I controlled the drugs the bills amounted to about £120. I may say that this difference surprised me exceedingly, because, although I knew that I was saving a little here and a little there, my many other duties did not allow me to do anything like the amount of pharmaceutical work which a proper dispenser could have done. On looking back I have no hesitation in saying that the drug accounts of this hospital could have been reduced to well below £100 a year by ordinary care in prescribing, and by putting in a very few hours' pharmacy a week.

The house surgeon need not regard dispensing as a bore or a waste of time; on the contrary, he will find, as I have done, that it is a most useful experience, and when he goes into general practice he will be able to do his duty faithfully to his patients, and yet spend less on drugs than many other practitioners.

I will not now enter into details as to how economy in this direction may be effected; but may be allowed to point out that if such a saving as above mentioned can be made by a house surgeon, who naturally has to keep in stock everything likely to be ordered by the staff (and you may be sure that serums, potassium iodide, and new preparations were not stinted), how much easier must it be for a general practitioner to exercise economy if he knows how to buy and how to dispense! It must not be thought that I am an advocate of the "mag. sulph. and sacch. ust." style of prescribing, nor do I believe in cheap drugs; on the contrary, I am a great believer in the use of standardised preparations.

